# Post-COVID-19 human memory impairment: A PRISMA-based systematic review of evidence from brain imaging studies

**DOI:** 10.3389/fnagi.2022.1077384

**Published:** 2022-12-09

**Authors:** Dan Shan, Shaoyang Li, Ruichen Xu, Glen Nie, Yangyiran Xie, Junchu Han, Xiaoyi Gao, Yuandian Zheng, Zhen Xu, Zhihao Dai

**Affiliations:** ^1^Department of Biobehavioral Sciences, Columbia University, New York, NY, United States; ^2^Faculty of Science, The Hong Kong Polytechnic University, Kowloon, Hong Kong SAR, China; ^3^Department of Integrative Biology, University of Wisconsin-Madison, Madison, WI, United States; ^4^Department of Biological Science, Northeastern University, Boston, MA, United States; ^5^School of Medicine, Vanderbilt University, Nashville, TN, United States; ^6^New York State Psychiatric Institute, Global Psychiatric Epidemiology Group, New York, NY, United States; ^7^School of Medicine, Saint Louis University, St. Louis, MO, United States; ^8^Minhang Crosspoint Academy at Shanghai Wenqi Middle School, Shanghai, China; ^9^School of Medicine, Royal College of Surgeons in Ireland, University of Medicine and Health Sciences, Dublin, Ireland

**Keywords:** brain, COVID-19, memory impairment, neuroimaging, PET, MRI

## Abstract

Many people with coronavirus disease 2019 (COVID-19) report varying degrees of memory impairment. Neuroimaging techniques such as MRI and PET have been utilized to shed light on how COVID-19 affects brain function in humans, including memory dysfunction. In this PRISMA-based systematic review, we compared and summarized the current literature looking at the relationship between COVID-19-induced neuropathological changes by neuroimaging scans and memory symptoms experienced by patients who recovered from COVID-19. Overall, this review suggests a correlational trend between structural abnormalities (e.g., cortical atrophy and white matter hyperintensities) or functional abnormalities (e.g., hypometabolism) in a wide range of brain regions (particularly in the frontal, parietal and temporal regions) and memory impairments in COVID-19 survivors, although a causal relationship between them remains elusive in the absence of sufficient caution. Further longitudinal investigations, particularly controlled studies combined with correlational analyses, are needed to provide additional evidence.

## Introduction

It is not uncommon for patients who recovered from coronavirus disease-2019 (COVID-19) to experience memory impairment in conjunction with other systemic or respiratory symptoms (Alonso-Lana et al., 2020; Huang et al., 2020; Ahmed et al., 2022). Specifically, short-term and long-term neurological and neuropsychiatric changes were observed after the onset of COVID-19 in patients (Dasgupta et al., 2020; Beghi et al., 2021; Roy et al., 2021), possibly leading to difficulties with memory (Al-Ramadan et al., 2021; Llach and Vieta, 2021). Ahmed et al. found that approximately one in five patients with varying severity of COVID-19 experienced memory difficulties during their recovery period within 1 year (Ahmed et al., 2022). Søraas et al. noted that about 10 percent of mild and non-hospitalized patients still had memory complaints 8 months after recovery from COVID-19 (Søraas et al., 2021).

Recent studies have provided evidence for the etiology of memory deficit caused by COVID-19 (Cataldi et al., 2020; Iadecola et al., 2020; Koralnik and Tyler, 2020; Wu et al., 2020; Najt et al., 2021). For example, a reduction in the gray matter volume such as the frontal lobe, which is responsible for working memory capacity, has been reported in certain COVID-19 patients (Prabhakaran et al., 2000; Lu et al., 2020; Douaud et al., 2021). The hippocampus, important for especially episodic and spatial memory (Scoville and Milner, 1957; Parkin, 1996; Eichenbaum, 2001), has emerged as a particularly vulnerable region affected by the SARS-CoV-2 virus. The virus could travel along peripheral olfactory neurons (Han et al., 2020; Soler et al., 2020; Kay, 2022), and invade the hippocampus through rapid interneuronal trans-synaptic viral dissemination within brain cortical regions (Kumar et al., 2020; Lisianiy and Pedachenko, 2022), potentially resulting in impaired memory after infection (Ritchie et al., 2020). Furthermore, the damage in the hippocampus could be further amplified by the immune response against SARS-CoV-2 virus, which could affect the blood-brain barrier (BBB) (Achar and Ghosh, 2020; Morris and Zohrabian, 2020). In addition, COVID-19 has been reported to cause silent brain hypoxia (Somers et al., 2020; Garg et al., 2021; Rahman et al., 2021), which also contributes to the hippocampal damage (Ahmed et al., 2022).

These findings raise three questions. First, how do findings on the effects of SARS-CoV-2 virus on the brain at the tissue, cellular, and molecular levels relate to the clinical and structural brain characteristics of individuals infected with COVID-19? Second, could self-report symptoms or memory tests truly reflect the memory impairment of COVID-19 survivors? Third, what types of memory problems are unique to COVID-19 patients? Answering these questions is critical to interpreting the relationship between COVID-19 and memory impairment. To answer the first question, although brain-related pathologies regarding COVID-19 were well interpreted in previous studies, memory deficits associated with COVID-19 are still being explored. There is mixed evidence of hippocampal volume change post-COVID-19. Mahajan and Mason pointed out that the reduction in hippocampal volume could indicate cognitive deficits in memory (Mahajan and Mason, 2021), and this has been reported in COVID-19 patients (Liu et al., 2022). Similarly, decreased neurogeneration due to COVID-19-related neuroinflammation in the hippocampus could result in cognitive decline, including memory loss (Lynch, 2010; Bayat et al., 2022; Radhakrishnan and Kandasamy, 2022). In contrast, a follow-up study looking at both self-report and brain imaging data showed that COVID-19 survivors had significantly greater structural volume and functional activity in the hippocampus bilaterally as compared with healthy controls (Tu et al., 2021). Although memory function was not directly assessed in these COVID-19 survivors (Tu et al., 2021), a larger hippocampal volume is considered to be associated with better memory capability (Biegler et al., 2001; Pohlack et al., 2014). To answer the second question, participants' self-reported outcomes (i.e., subjective memory complaints assumed to arise due to COVID-19 infection) have limited reliability (e.g., Hampshire et al., 2021; Søraas et al., 2021). Memory test is more objective than self-report, but still cannot provide definitive evidence of neuropathological changes associated with memory impairment. To answer the third question, brain imaging data can help monitor structural and functional changes in brain regions implicated in memory (Weiner and Khachaturian, 2005; Health Quality Ontario, 2014; Morita et al., 2016). This can also be used in evaluating asymptomatic COVID-19 patients (Samkaria and Mandal, 2021). However, the diagnostic value of various brain scans regarding memory impairment in COVID-19 patients is unclear.

Memory plays an enormous role in a person's daily living (Cohen, 2008), therefore, access to the post-COVID-19 memory dysfunction is crucial. Direct evidence of memory dysfunction may come from brain imaging studies in COVID-19 survivors, but little review has been done in this field. In this systemic review, we intended to present the most up-to-date information on memory dysfunction in COVID-19 survivors by collecting previous neuroimaging evidence.

## Methods

The current systematic review was carried out based on the guidelines and principles outlined by the Preferred Reporting Items for Systematic Reviews and Meta-Analyses (PRISMA) statement 2020 and checklist (Page et al., 2021).

We performed a search through the PubMed database for relevant studies published in English from Jan 1, 2020, to September 7, 2022. The reference lists of all original articles and reviews retrieved from the search were subjected to manually review to identify additional studies that may fit the systematic review objective in the current article but were not identified by the PubMed database. Following keywords were used in our search strategy: (“memory complaint” OR “memory deficit” OR “memory impairment” OR “memory loss”), AND (“COVID-19” OR “COVID pandemic”), AND (“brain imaging” OR “magnetic resonance imaging” OR “MRI” OR “functional magnetic resonance imaging” OR “fMRI” OR “positron emission tomography” OR “PET” OR “electroencephalography” OR “EEG” OR “magnetoencephalography” OR “MEG” OR “event related potential” OR “ERP” OR “diffusor tensor imaging” OR “DTI” OR “SPECT” OR “CT”).

We set inclusion criteria that primarily specified original peer-reviewed research articles, in which case, only original empirical articles were retained when any systematic or narrative review that also discussed the neuroimaging findings regarding memory in COVID-19 patients was identified. We screened all relevant studies that recruited COVID-19 patients and utilized *in-vivo* brain-imaging modalities. We retained studies that reported neuroimaging findings regarding memory in patients with COVID-19; studies that examined the memory function of participants only through memory tests or self-reported results were excluded; case series or case reports were also systematically reviewed and discussed, but separately from the original articles.

Apart from clinical imaging data that could be relevant to the brain memory function of COVID-19 participants, the data extracted from each article included brain-imaging modality. In addition, where possible, we calculated effect sizes (Cohen's d for differences between means) for the purpose of comparisons (Larner, 2014; Mouchlianitis et al., 2016).

## Results

**Figure 1:**
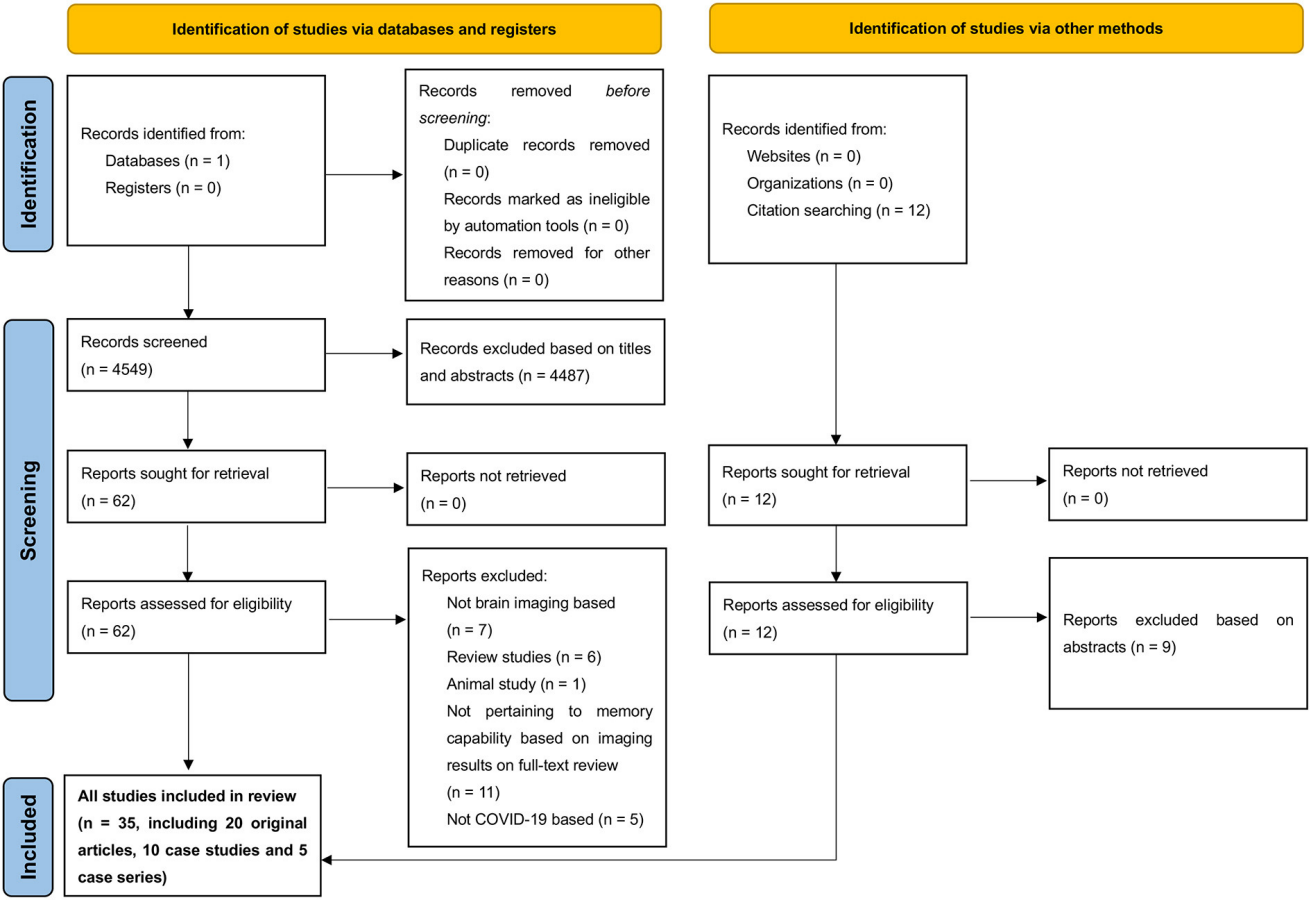
Preferred Reporting Items for Systematic Reviews and Meta-Analyses (PRISMA) diagram demonstrating search strategy.

We identified 4,561 reports, 35 of which fulfilled the inclusion criteria after the full texts were assessed (Figure 1) (Garg et al., 2020; Kushwaha et al., 2020; Lu et al., 2020; Sun et al., 2020; Vandervorst et al., 2020; Woo et al., 2020; Blazhenets et al., 2021; Branco de Oliveira et al., 2021; Donegani et al., 2021; Ermis et al., 2021; Guedj et al., 2021; Hellgren et al., 2021; Hosp et al., 2021; Jacobs et al., 2021; Kas et al., 2021; Matias-Guiu et al., 2021; Polascik et al., 2021; Ravaglia et al., 2021; Silva et al., 2021; Sollini et al., 2021; Yesilkaya et al., 2021; Allen and Absar, 2022; Bungenberg et al., 2022; Cecchetti et al., 2022; Das et al., 2022; Douaud et al., 2022; Dressing et al., 2022; Hadad et al., 2022; Huang C. et al., 2022; Huang S. et al., 2022; Hugon et al., 2022a,b; Martini et al., 2022; Morand et al., 2022; Savino et al., 2022; Terruzzi et al., 2022). All the 35 studies assessing brain anatomical or functional changes in patients following SARS-CoV-2 infection discussed the impacts of COVID-19 on memory function. 20 studies were classified as original articles, 10 as case studies, and 5 as case series.

These included studies were deemed of sufficient methodological qualities, in terms of the JBI Critical Appraisal Checklists (and the mean percentage scored points 97.3%) (Barker et al., 2022), though some were of relatively lower quality. The main missing quality components are as follows: The criteria of “consecutive inclusion of participants” was absent in two case series studies (Hugon et al., 2022b; Savino et al., 2022); of “control group” was absent in one prospective cohort study (Hellgren et al., 2021); of “identified confounding factors” was absent in three studies (Sun et al., 2020; Guedj et al., 2021; Hadad et al., 2022); And, of “strategies to deal with confounding factors” was absent in three studies (Ermis et al., 2021; Hellgren et al., 2021; Bungenberg et al., 2022). Refer to Table 1 for a summary of the quality of the included studies.

**Table 1:** Summarized quality assessment of the included articles.

**Articles**	**Quality assessment**
Allen and Absar, 2022	8/8 (100.0%)^a^
Blazhenets et al., 2021	11/11 (100.0%)^b^
Branco de Oliveira et al., 2021	8/8 (100.0%)^a^
Bungenberg et al., 2022	7/8 (87.5%)^c^
Cecchetti et al., 2022	11/11 (100.0%)^b^
Das et al., 2022	8/8 (100.0%)^a^
Donegani et al., 2021	8/8 (100.0%)^c^
Douaud et al., 2022	10/10 (100.0%)^d^
Dressing et al., 2022	11/11 (100.0%)^b^
Ermis et al., 2021	7/8 (87.5%)^c^
Garg et al., 2020	8/8 (100.0%)^a^
Guedj et al., 2021	9/10 (90.0%)^d^
Hadad et al., 2022	7/8 (87.5%)^c^
Hellgren et al., 2021	9/11 (81.8%)^b^
Hosp et al., 2021	11/11 (100.0%)^b^
Huang S. et al., 2022	8/8 (100.0%)^c^
Hugon et al., 2022a	10/10 (100.0%)^e^
Hugon et al., 2022b	9/10 (90.0%)^e^
Jacobs et al., 2021	8/8 (100.0%)^a^
Kas et al., 2021	11/11 (100.0%)^b^
Kushwaha et al., 2020	8/8 (100.0%)^c^
Lu et al., 2020	11/11 (100.0%)^b^
Martini et al., 2022	11/11 (100.0%)^b^
Matias-Guiu et al., 2021	8/8 (100.0%)^a^
Morand et al., 2022	10/10 (100.0%)^e^
Polascik et al., 2021	10/10 (100.0%)^e^
Ravaglia et al., 2021	8/8 (100.0%)^a^
Savino et al., 2022	9/10 (90.0%)^e^
Silva et al., 2021	8/8 (100.0%)^c^
Sollini et al., 2021	10/10 (100.0%)^d^
Sun et al., 2020	10/11 (90.9%)^b^
Terruzzi et al., 2022	8/8 (100.0%)^a^
Vandervorst et al., 2020	8/8 (100.0%)^a^
Woo et al., 2020	8/8 (100.0%)^c^
Yesilkaya et al., 2021	8/8 (100.0%)^a^

a According to the Joanna Briggs Institute (JBI) critical appraisal checklist for case study.

b According to the Joanna Briggs Institute (JBI) critical appraisal checklist for cohort studies.

c According to the Joanna Briggs Institute (JBI) critical appraisal checklist for analytical cross-sectional studies.

d According to the Joanna Briggs Institute (JBI) critical appraisal checklist for case-control studies.

e According to the Joanna Briggs Institute (JBI) critical appraisal checklist for case series.

Brain scans were implemented through multiple neuroimaging techniques: 23 studies used MRI, 15 used PET, 9 used EEG, 8 used CT, 3 used DTI, and 1 used MRS (see Tables 2, 3).

**Table 2:** Summary of study procedures and findings of included original articles.

**Included studies**	**Modalities**	**Study procedures and main findings**
Blazhenets et al., 2021	PET	Follow-up ^18^F-FDG PET revealed that the initial significant hypometabolism of the frontoparietal and temporal cortical regions improved over time, accompanied by recovered memory function. However, memory impairment and neocortical hypometabolism still existed after 6 months of COVID-19 recovery in some patients.
Bungenberg et al., 2022	Structural MRI	38% COVID-19 patients with Long COVID had memory complaints. Brain microbleeds were found exclusively in hospitalized patients. Brain atrophy and white matter hyperintensities were not found significantly different between hospitalized and non-hospitalized COVID-19 patients.
Cecchetti et al., 2022	Structural MRI and EEG	Over one third of patients with a recent COVID diagnosis suffered memory problems. Compared to healthy controls, these patients showed higher regional current density and connectivity at delta band, correlating with executive performances, and greater white matter hyperintensities (*d* = 0.47), correlating with verbal memory deficits. Lower EEG delta band at baseline predicted worse memory capability at follow-up, though the EEG findings at 10 months were improved in most patients. Dysgeusia and hyposmia during the acute phase of COVID-19 were linked with increased vulnerability in patients' memory functions over time.
Donegani et al., 2021	PET	COVID-19 patients complained of persistent olfactory dysfunction, which is very likely to impact olfactory memory (relevant for working memory). ^18^F-FDG PET results showed hypometabolism in parahippocampal fusiform gyri in both hemispheres and left insula in those with hyposmia.
Douaud et al., 2022	Structural MRI	Compared to healthy controls, MRI results showed greater reductions in gray matter thickness in COVID-19 patients bilaterally especially in the parahippocampal gyrus, anterior cingulate cortex and temporal pole as well as a greater reduction in global brain size in the COVID-19 patients.
Dressing et al., 2022	PET	All patients (100%) complained about memory problems. However, no significant changes of regional cerebral glucose metabolism were shown.
Ermis et al., 2021	EEG	EEG showed diffuse pathological slowing and intermittent rhythmic delta-activity in two COVID-19 patients with severe short-term memory deficit (delayed recall) in the Montreal Cognitive Assessment (MoCA).
Guedj et al., 2021	PET	Nearly 50% of long-COVID-19 patients experienced memory impairments. In comparison to healthy participants, these patients had bilateral hypometabolism in the bilateral rectal/orbital gyrus; the right temporal lobe, including the amygdala and the hippocampus, extending to the right thalamus; the bilateral pons/medulla brainstem; the bilateral cerebellum (p-voxel < 0.001 uncorrected, p-cluster < 0.05 FWE-corrected).
Hadad et al., 2022	Structural MRI, CT and EEG	All patients reported their memory impairments. However, MRI, CT, and EEG did not show alternative etiologies for the memory complaints reported in these patients (such as cerebrovascular disease, inflammatory disorder and tumor).
Hellgren et al., 2021	Structural MRI	Short-term memory deficit was the most prevalent (25%) cognitive impairments of COVID-19 patients. MRI showed multiple subcortical white matter lesions in over 70% of patients, particularly in the frontal and parietal lobes.
Hosp et al., 2021	Structural MRI and PET	Among all cognitive domains of MoCA memory was most severely affected in subacute COVID-19 patients. MRI showed no sign of cerebral atrophy. ^18^F-FDG PET revealed pathological results in most patients with predominant frontoparietal hypometabolism, which was highly correlated (*R*^2^ = 0.62) with MoCA performance.
Huang S. et al., 2022	DTI	No significant difference about working memory capability was found between recovered COVID-patients and healthy controls, or between ICU and non-ICU patients. DTI results showed that COVID-19 patients who were admitted to the intensive care unit had slightly more white matter abnormalities; and that white matter abnormalities of all patients were improved over time, with those with shorter hospital stays improving more quickly.
Kas et al., 2021	Structural MRI and PET	MRI showed no specific abnormalities in white matter in patients with memory deficits. During the acute phase of COVID-19 infection, PET showed significant hypometabolism of bilateral prefrontal cortex (*d =* 1.98), anterior cingulate cortex (*d =* 1.81), middle temporal gyrus (*d =* 1.99), inferior temporal gyrus (*d =* 1.53), caudate nucleus (*d =* 2.01), cerebellar hemispheres (*d =* 1.52), and insula (*d =* 2.11) in these patients. These hypometabolisms improved and was accompanied with improvement of memory over time.
Kushwaha et al., 2020	EEG and Structural MRI	7% of COVID-19 patients had memory impairment. MRI showed T2/Flair hyperintensity in left temporo-occipital lobe, hippocampus with diffusion restriction, and right frontal periventricular white matter T2 flair hyperintensity. EEG revealed generalized slowing.
Lu et al., 2020	Structural MRI and DTI	During COVID-19 infection, over 13% of patients complained of memory loss. MRI and DTI screenings performed 3 months later showed significantly increased global gray matter volume (GMV), GMVs in left Rolandic operculum, right cingulate, bilateral hippocampi, left Heschl's gyrus, and decreased global mean diffusivity of white matter in COVID-19 patients with memory loss, as compared to healthy controls (*p* < 0.05).
Martini et al., 2022	PET	^18^F-FDG PET revealed that severe brain hypometabolism affected nearly all cortical regions such as the hippocampus and amygdala in the acute phase of COVID-19 in patients. But brain hypometabolism was no longer detected in 7–9 months after the onset of COVID-19. In COVID-19 patients with memory difficulties, PET also suggested that the extension and severity of brain hypometabolism was significantly and negatively correlated with immediate memory performance.
Silva et al., 2021	DTI	Two months after the onset of COVID-19, 33% of patients still subjectively reported persistent memory difficulties. DTI results revealed no significant correlation between the logical memory scores (immediate and delayed recall) and diffusivities in the parahippocampal cinguli. The reduced fractional anisotropy in the acute phase of COVID-19 infection may suggest overall white matter damage.
Sollini et al., 2021	PET	No long COVID patients out of 13 complained of memory deficits as one of their persistent symptoms (for at least 30 days after infection recovery) at the time of ^18^F-FDG brain PET scan, which revealed their brain hypometabolism in the right parahippocampal gyrus and thalamus (uncorrected *p* < 0.001 at voxel level).
Sun et al., 2020	CT	Around 40% of COVID-19 patients with an average age of 77 experienced memory impairments. Memory symptoms would be more severe in those with more brain atrophy and cerebral ischemia revealed by brain CT, or with more serious COVID-19 infection, or with other neuropsychiatric diseases.
Woo et al., 2020	Structural MRI	Over 40% of post-COVID-19 patients experienced short-term memory deficits. Cranial MRI utilized in two severely affected patients showed no structural abnormalities leading to their memory impairments.

Effect sizes (Cohen's d for differences between means) are shown where the calculations were possible.

CT, Computed Tomography; DTI, Diffusion Tensor Imaging; EEG, electroencephalogram; FDG, fluorodeoxyglucose; GMV, Gray Matter Volume; MRI, Magneti Resonance Imaging; PET, Positron Emission Tomography.

**Table 3:** Summary of study procedures and findings of included case studies and case series.

**Included studies**	**Modalities**	**Study procedures and main findings**
Allen and Absar, 2022^*^	PET and Structural MRI	A 79-year-old female patient without significant past medical history complained mild short-term memory loss over several years likely due to aging. This memory impairment did not interfere with her daily life until her confirmed diagnosis of COVID-19, which severely worsened her dementia especially episodic memory and word finding difficulties over 10 months following COVID-19 infection. No abnormalities were seen on her brain MRI. PET scan exhibited obvious hypometabolism in the left temporal lobe and mild hypometabolism in the adjacent left frontal and parietal regions.
Branco de Oliveira et al., 2021^*^	PET and Structural MRI and EEG	A 47-year-old female patient experienced persistent short-term memory problems during COVID infection. Brain MRI and EEG exams showed no abnormalities. Her memory impairment improved after 2 days of treatment during hospitalization. Complaint about discrete memory still existed after 20 days from discharge, though it did not interfere with her daily life activity.
Das et al., 2022^*^	Structural MRI and EEG	Cognitive function assessment noted impairment of recent memory in a 33-year-old male patient with no significant past medical or psychiatric history following COVID infection. Brain MRI scan showed few tiny and non-specific hyperintense foci in bilateral frontal subcortical and left parietal periventricular white matter. EEG showed intermittent diffuse slow waves with atypical triphasic waves suggestive of diffuse dysfunction of brain.
Garg et al., 2020^*^	CT and Structural MRI	A 59-year-old female patient complained about memory deficits (difficulty remembering daily schedules and names) after initial COVID infection. When her memory impairments acutely worsened 2 months after COVID infection, she was found to have a chronic ischemic stroke. CT and MRI of the head were carried out during an emergency admission 2 months after her initial viral infection. Non-contrast CT suggested hypodensity in the left frontal lobe, while MRI with contrast indicated hyperintense signal abnormalities in the left frontal lobe.
Hugon et al., 2022a^**^	PET and Structural MRI	Fluorodeoxyglucose PET was performed in three COVID-19 patients with cognitive complaints, especially with memory problems as shown in the complete neuropsychological examinations. Brain MRIs were normal in all three patients. The hypometabolic pons was observed in these patients, as revealed by the cerebral FDG PET.
Hugon et al., 2022b^**^	PET	Cerebral FDG PET showed significant brainstem hypometabolism, especially in the pons in three COVID-19 patients with memory impairments.
Jacobs et al., 2021^*^	MRI and CT	A 55-year-old male patient with COVID-19 suffered memory impairment. Brain CT showed hypodense ischemic plaques extending from the lentiform nucleus to the anterior horn of the left lateral ventricle. MRI scan showed zones with diffusion restriction in the bilateral corona radiata extending up to the semioval regions.
Matias-Guiu et al., 2021^*^	PET and Structural MRI	A 67-year-old female patient without past medical or psychiatry history developed obvious memory problems 6 months after the onset of COVID-19. One month later, the neuropsychological assessments suggested verbal episodic memory deficits. MRI and PET showed no abnormalities on visual analysis, but semi-quantitative analysis of meta-region of interest (meta-ROI) of FDG-PET indicated hypometabolism in regions associated with Alzheimer's Disease (such as the hippocampal region).
Morand et al., 2022^**^	PET	[^18^F]-FDG PET metabolism of seven pediatric patients with suspected long-COVID was evaluated in comparison to 21 pediatric controls, and of 35 adult patients with long COVID in comparison to 44 healthy adult controls. It was found that the seven pediatric patients experienced memory difficulties for more than 4 weeks following the initial acute COVID-19 phase, without symptom-free interval. After an average of 5 months, pediatric patients showed a pattern of brain hypometabolism similar to that seen in adults with long-COVID, involving bilateral medial temporal lobes (such as amygdala and parahippocampal gyrus), brainstem, and cerebellum (p-voxel < 0.001, p-cluster < 0.05 FWE-corrected).
Polascik et al., 2021^**^	Structural MRI and CT	Three adult patients over age 65 complained significantly worsened memory difficulties after COVID-19 infection. Non-contrast head CT before COVID-19 infection showed mild bilateral hippocampal atrophy in patient 1; MRI in patient 2 revealed mild hippocampal and biparietal lobe atrophy as well as mild cerebral white matter disease; non-contrast head CT performed 3 weeks after COVID-19 diagnosis showed calcification in the right basal ganglia and a mild hypodense signal periventricularly in patient 3. Noncontrast brain MRI showed subtle hippocampal and perisylvian atrophy and mild to moderate parietal lobe atrophy bilaterally.
Ravaglia et al., 2021^*^	MRI	A 58-year-old female patient without any past medical condition experienced transient global amnesia (TGA), spatial memory in particular, 1 h after executing first-time PCR viral swab test for COVID-19 (later revealing as negative). MRI was performed 1 day after TGA. DWI image suggested a typical punctate focus restriction in her right hippocampus. However, the swab-induced physical or psychological stimulation was believed the trigger of causing her hippocampal abnormality.
Savino et al., 2022^**^	Structural MRI, EEG and CT	Five patients under 18 years of age were admitted due to new-onset neuropsychiatric symptoms. In case 1, a 15-year-old male patient suffered recent memory impairment in the absence of gross cognitive decline. Brain CT, EEG and MRI showed no significant abnormalities, except for a slight descent of the right cerebellar tonsil and a thinning corpus callosum. Both EEG and MRI were normal in other four cases, who did not report memory problems.
Terruzzi et al., 2022^*^	Structural MRI, PET and EEG	A 51-year-old female patient complained episodic and prospective memory problems, which were confirmed *via* neuropsychological examination. EEG results were normal, but MRI scan showed hyperintensity of signal in the paracallosal and periventricular white matter. The ^18^F-FDG PET-CT brain scan following her hospital discharge showed cortical hypometabolism, except for the occipital lobes.
Vandervorst et al., 2020^*^	Structural MRI and CT	Cognitive testing revealed immediate and short-term memory deficits in a 29-year-old male patient with no prior medical history 2 weeks after the onset of COVID-19 symptoms CT scan was normal, while the first MRI scan showed an asymmetric FLAIR (fluid-attenuated inversion recovery) hyperintensity in the left medial temporal cortex associated with mild gyral expansion. During his hospitalization, a second brain MRI scan, 4 days after the first MRI, showed normalization of cortical hyperintensity and gyral expansion; meanwhile, his memory difficulties improved.
Yesilkaya et al., 2021^*^	Structural EEG, CT, and MRI and MRS	A 29-year-old male patient developed memory impairment, such as difficulty recalling past experiences 2 weeks after the completion of treatment for COVID-19. FAB was used to evaluate frontal lobe dysfunction and GDS was used to stage cognitive decline. The FAB score was 13 and the GDS stage was 3 initially, indicating mild memory decline. No memory deficit was detected after 3 months. Structural neuroimaging results through EEG, CT and MRI were normal in his initial screening. MRS of the bilateral DLPFC 1 week after COVID infection revealed significantly decreased levels of N-acetylaspartate (166,65), glutamate (31,54), and glutamate/glutamine ratio (0,13). Three months later, a repeat MRS of DLPFC suggested significantly improved levels of N-acetylaspartate (183,14), while levels of the other two metabolites did not improve compared to the first MRS.

CT, Computed Tomography; DLPFC, dorsolateral prefrontal cortex; DTI, Diffusion Tensor Imaging; EEG, electroencephalogram; FAB, Frontal Assessment Battery; FDG, fluorodeoxyglucose; GDS, Global Deterioration Scale; GMV, Gray Matter Volume; MRI, Magnetic Resonance Imaging; MRS, MR-spectroscopy; PET, Positron Emission Tomography.

* Case study.

** Case series.

Among the 20 original studies included, findings suggested that the frequency of memory impairment ranged from 0 to 100%, with a mean average of more than 30% across studies, subjected to the age of the participants, past medical history, the recovery time after COVID-19 infection, severity of COVID-19 symptoms, and type of memory impairment (e.g., short-term vs. long-term memory) (Kushwaha et al., 2020; Lu et al., 2020; Sun et al., 2020; Woo et al., 2020; Blazhenets et al., 2021; Donegani et al., 2021; Ermis et al., 2021; Guedj et al., 2021; Hellgren et al., 2021; Hosp et al., 2021; Kas et al., 2021; Silva et al., 2021; Sollini et al., 2021; Bungenberg et al., 2022; Cecchetti et al., 2022; Douaud et al., 2022; Dressing et al., 2022; Hadad et al., 2022; Huang C. et al., 2022; Huang S. et al., 2022; Martini et al., 2022). COVID-19 patients were much more likely to experience short-memory deficits compared to long-term memory. Memory impairment could be more severe in those with other neurological conditions such as cerebral ischemia. In terms of brain scan results, all ^18^F-FDG PET scans, with the exception of the study by Dressing et al. (2022), showed initially a markedly widespread hypometabolism after COVID-19 infection, which may be found in the prefrontal, frontoparietal and temporal regions (e.g., amygdala and hippocampus), thalamus, pons/medulla brainstem, and cerebellum. Over time, most of the affected brain regions of the patient's hypometabolism improved, in most cases accompanied by a substantial improvement in memory function. MRI results showed inconsistent findings: around 50% of MRI scans revealed that no significant brain pathological changes (e.g., brain atrophy or white matter hyperintensities) were associated with COVID-19 that caused patients' memory problems; whilst the other 50% of MRI scans showed white matter hyperintensities (particularly in the frontal and parietal lobes) and significant reductions in gray matter thickness bilaterally (especially in the parahippocampal gyrus, anterior cingulate cortex and temporal pole) in COVID-19 patients, as compared to healthy controls; although Lu et al. found that COVID-19 patients had higher global gray matter volume than healthy controls 3 months after infection with COVID-19 (Lu et al., 2020). EEG scans showed that diffuse pathological slowing, intermittent rhythmic delta-activity and low delta band at baseline were associated with memory impairment in COVID-19 patients. All three DTI studies indicated white matter abnormalities in COVID-19 patients, especially during the acute phase of infection (Lu et al., 2020; Silva et al., 2021; Huang C. et al., 2022; Huang S. et al., 2022). Although this might be biased due to the limited previous studies carried out utilizing DTI as references, the high sensitivity of DTI to brain white matter damage has been demonstrated (Kiely et al., 2022). Despite Lu et al. found increased white matter integrity in COVID-19 patients 3 months after infection, compared to healthy controls, Lu et al. attributed this to the underlying intrinsic reconstruction processes of brain white matter tracts (e.g., remyelination), which occurred following infection (Cauley and Cataltepe, 2014; Lu et al., 2020) (see Table 2 for details).

In the 15 case studies and case series included, findings suggested that COVID-19 patients in all age groups (from 10 to 79 years) in the absence of significant past medical or neuropsychiatric conditions could still suffer memory problems after the onset of COVID-19 (Garg et al., 2020; Vandervorst et al., 2020; Branco de Oliveira et al., 2021; Jacobs et al., 2021; Matias-Guiu et al., 2021; Polascik et al., 2021; Ravaglia et al., 2021; Yesilkaya et al., 2021; Allen and Absar, 2022; Das et al., 2022; Hugon et al., 2022a,b; Morand et al., 2022; Savino et al., 2022; Terruzzi et al., 2022). These patients' self-reported or cognitive function assessment-confirmed memory deficits may be in the form of short-term memory or long-term memory, or both. Memory symptoms could appear quickly after the initial onset of COVID-19, and may persist for more than 10 months, although they could be improved over time during recovery phases in most patients. The severity of memory symptoms could be worse in elderly patients, especially in the case that they were having other neurological conditions, such as stroke elicited by the consequences of COVID-19. In terms of brain imaging results, brain abnormalities in patients after the onset of COVID-19 might be reflected in the hypometabolism in bilateral medial temporal lobes (e.g., amygdala, parahippocampal gyrus), adjacent left frontal and parietal regions, brainstem (especially the pons) and cerebellum, as shown by PET scans; in the hyperintensities of white matter in various regions (e.g., left frontal lobe, paracallosal and periventricular regions), as well as hippocampal atrophy and biparietal lobe atrophy, as revealed by structural MRI; in the intermittent diffuse slow waves with atypical triphasic waves, as EEG showed; in the hypodensity in the left frontal lobe and periventricular area, as CT showed; and in the significantly decreased levels in N-acetylaspartate, glutamate and glutamate/glutamine ratio in bilateral dorsolateral prefrontal cortex, as MRS showed; although these abnormalities could be ameliorated in follow-up evaluations (see Table 3 for details).

## Discussion

This systematic review revealed that either structural abnormalities (e.g., cortical atrophy and white matter hyperintensities) or functional abnormalities (e.g., hypometabolism) in widespread brain regions (particularly in the frontal, parietal and temporal regions) may exist in COVID-19 patients with memory impairment compared to healthy controls. These brain abnormalities and memory dysfunction were likely to be reversible over time in most cases. The direction (i.e., increase vs. decrease) of the anatomical and metabolic alterations initially was in line with imaging findings in patients with comparable memory impairments such as dementia and Alzheimer's disease (Meyer et al., 2022).

To understand the association between COVID-19 and ensuing memory impairment in the human brain, three key questions need to be considered. The first question is, which certain brain regions are highly responsible for memory functions? The second question is that whether these regions are significantly affected by COVID-19? The third question is that whether there is a threshold for the severity of COVID-19 infection to elicit memory symptoms in patients? For the last question, Ahmed et al. found that COVID-19 severity was independent of patients' memory impairments (Ahmed et al., 2022), competing with the results of other studies (e.g., Sun et al., 2020; Méndez et al., 2021). All these studies shared the consensus that memory complaints may still occur in patients with mild COVID-19, suggesting that there was no obvious threshold of COVID-19 severity inducing memory complaints. For the first and second questions, from a neuroscience perspective, the fronto-parietal brain regions are important for short-term memory (e.g., working memory) (Chai et al., 2018; Assem et al., 2020), and the temporal region for long-term memory (Hershey et al., 1998; Simons and Spiers, 2003; Zokaei et al., 2019). All these regions are highly susceptible to COVID-19 infection in patients, especially during the early post-infection phase (Egbert et al., 2020; Douaud et al., 2022; Oumerzouk et al., 2022; Zalpoor et al., 2022). Thus, it is possible for COVID-19 infection to cause brain damage and subsequent memory dysfunction in patients.

To better understand this relationship between COVID-19 infection and memory dysfunction, another key point to concern is what the specific neurological anatomical or functional manifestations of COVID-19 infection are. Although impaired memory functions in COVID-19 patients are probably associated with the brain abnormalities as shown in the previous neuroimaging findings, a direct causal relationship cannot be established without sufficient caution.

Structural MRI and ^18^F-FDG PET were the most frequently utilized imaging scans in the previous studies investigating brain changes associated with memory impairment. Of note, these studies did not disclose fully consistent findings. For MRI research, not all studies using structural MRI/CT techniques found consistent structural brain abnormalities in COVID-19 patients with memory impairment. For example, Hosp et al. suggested that no sign of cerebral atrophy was seen in subacute COVID-19 patients with memory impairment (Hosp et al., 2021). Hospitalized COVID-19 patients were found to have worse verbal memory compared to non-hospitalized COVID-19 patients; however, no significant difference was found in terms of brain atrophy and white matter hyperintensities between the two groups (Bungenberg et al., 2022). Kas et al. suggested that no specific abnormalities in white matter in COVID-19 patients with memory deficits were found (Kas et al., 2021). Particularly, Lu et al. proposed that COVID-19 patients (including those with memory loss) had statistically significantly higher global gray matter volumes 3 months after COVID-19 infection, compared to non-COVID-19 controls (Lu et al., 2020), contrasting to many previous findings (Newhouse et al., 2022). In addition, 7 of 11 case studies or case series that utilized structural MRI scans also showed that very few abnormalities were seen in COVID-19 patients with memory dysfunction (Branco de Oliveira et al., 2021; Matias-Guiu et al., 2021; Yesilkaya et al., 2021; Allen and Absar, 2022; Das et al., 2022; Hugon et al., 2022a; Savino et al., 2022). In terms of PET research, although most previous studies using FDG-PET provided substantial evidence of hypometabolism in widespread brain regions (e.g., frontoparietal and temporal cortical regions) especially during the initial stage of COVID-19 infection, Dressing et al. found no significantly distinct changes of regional cerebral glucose metabolism in COVID-19 patients with memory complaints (Dressing et al., 2022).

There are several explanations for these inconsistent findings. First, differences in methods might yield conflicting findings, including the inclusion criteria for recruiting participants, follow-up duration of the studies, the approaches of determination of memory impairment (e.g., subjective self-report vs. objective memory assessment scores), the time points of memory symptoms reported and neuroimaging scans conducted, and data analysis methods. For instance, although Hosp et al. did not find cerebral structural abnormalities in COVID-19 patients using MRI, no correlational analysis between cerebral gray matter volume (or relevant) and memory performance was performed in those with memory symptoms; meanwhile, ^18^FDG PET imaging findings did show predominant frontoparietal hypometabolism, which was highly correlated with lower memory performance in COVID-19 patients (*R*^2^ = 0.62, *p* < 0.001) (Hosp et al., 2021). Similarly, although Kas et al. suggested that no specific abnormalities in white matter were seen on the MRI scan in COVID-19 patients with memory deficits, the PET scan showed widespread brain hypometabolism in these patients, especially during the acute phase of COVID-19 infection (Kas et al., 2021). Bungenberg et al. found hospitalized COVID-19 patients with worse memory capability had the similar brain atrophy and white matter hyperintensities as compared to non-hospitalized COVID-19 patients, which may indicate that brain pathoanatomical change was independent of COVID-19 severity in patients. Nevertheless, the correlational analysis of the relationship between memory performance and brain structural change was not conducted to better evaluate this association either in hospitalized patients or non-hospitalized ones (Bungenberg et al., 2022). Lu et al. found higher global gray matter volume in COVID-19 patients as compared to healthy controls. However, within the COVID-19 patients' cohort, global gray matter volume and reginal gray matter volume such as bilateral hippocampi were found to be negatively associated with memory loss in these patients (*r* = −0.341, −0.321, respectively; *p*-value = 0.008, 0.012, respectively) (Lu et al., 2020). Additionally, for the studies which did not conduct memory assessments *via* scales but relied solely on self-reported memory symptoms of patients to reflect their memory performance, there is a limitation regarding the quality (e.g., validity and generalizability of findings) of such research during the COVID-19 pandemic (Nieto et al., 2020). Second, Tian et al. pointed out that COVID-19 patients without the manifestations of memory deficits could still have brain pathological changes such as declined global cortical thickness. Hence, decreased cortical thickness (i.e., brain atrophy) might not be necessarily a direct cause of memory impairments in COVID-19 patients (Tian et al., 2022). In turn, it is possible that individuals with memory deficits had little apparent brain pathological changes in MRI scans as shown in the contrasting studies (Lu et al., 2020; Branco de Oliveira et al., 2021; Hosp et al., 2021; Kas et al., 2021; Matias-Guiu et al., 2021; Yesilkaya et al., 2021; Allen and Absar, 2022; Das et al., 2022; Hugon et al., 2022a; Savino et al., 2022). This may also partially explain the nuanced results in different studies. Finally, other confounding factors may also interfere with the relationship we are interested in exploring, including the types of memory symptoms (e.g., short-term vs. long-term memory, verbal vs. visual memory) manifested in the recruited participants, the recovery time (i.e., onset time of COVID-19) following infection, severity level of COVID-19, age of patients, past medical history, and etc.

From the perspective of neuroinflammatory-related changes in brain white matter, which are often reflected through DTI (Ji et al., 2017; Soni et al., 2021; Ouellette, 2022), all the three DTI studies included in this review suggested the abnormal changes in white matter in COVID-19 patients with memory symptoms (Lu et al., 2020; Silva et al., 2021; Huang C. et al., 2022; Huang S. et al., 2022), indicating the involvement of neuroinflammatory factors in these symptoms. Fernández-Castañeda et al. found that even mild COVID-19 infections could result in significant brain inflammation followed by brain cell dysregulation (e.g., loss of oligodendrocytes in white matter), and this would be expected to predict cognitive problems (Fernández-Castañeda et al., 2022a). Fernández-Castañeda et al. also proposed many similarities in inflammatory white matter damages and the pathophysiological processes of brain fog after COVID-19 as those after cancer chemotherapy. Evidence from both animal and postmortem showed that elevated inflammatory chemokines, especially CCL11, found in long COVID-19 patients with cognitive symptoms, directly contributed to the increased white matter microglial reactivity particularly in the hippocampus, an area highly responsible for learning and memory (Fernández-Castañeda et al., 2022a,b). These neuroinflammatory-related changes might be directly associated with early and transient memory impairment in COVID-19 patients.

In addition to the anatomical and physiological associations that have been discussed above, COVID-19 may also contribute to memory dysfunction by affecting patients' mental health (Amerio et al., 2020; Essadek and Rabeyron, 2020; Hyland et al., 2020; Shan et al., 2022a,b). This could also help explain the persistent memory symptoms in Long-COVID patients (Walia et al., 2021).

Since younger people were much more resilient against COVID-19 compared to the elderly (Bajaj et al., 2021), it is more reasonable that younger people infected with COVID-19 who subsequently self-reported memory impairment but with minimal neuroimaging changes actually experienced psychogenic causes such as brain fog, which could be elicited due to a stressful life event and ultimately led to their mild and temporary memory dysfunction, rather than triggered by direct and pronounced neural pathological changes on the brain (Loewenstein, 1993; Jennings et al., 2022). For example, Savino et al. noted a case of a 15-year-old male who presented with memory impairment, but his brain CT, EEG and MRI did not show obviously significant abnormalities (Savino et al., 2022). Hence, subjective self-reported memory symptoms at this time are more reflective of negative changes in memory performance in younger people than neuroimaging results. In contrast, the aging population were found to be more prone to evident brain pathological changes, such as reported evidence of accelerated amyloid formation of neurodegenerative proteins (de Erausquin et al., 2021; Semerdzhiev et al., 2021; Silva et al., 2022; Wu et al., 2022), so the objective Montreal Cognitive Assessment (MoCA) and neuroimaging results could better reflect the changes in these patients' memory performance. Thus, in future studies, we believe that subjects in different age groups should not be studied based on the same memory evaluation method in order to reflect actual changes in memory performance.

No permanent memory impairment induced by COVID-19 was demonstrated yet, and most memory symptoms are reversible over time. Tian et al. noted that the decrease in cortical thickness in COVID-19 patients improved and returned to baseline 10 months after discharge (regardless of COVID-19 severity), with no significant difference from healthy controls (Tian et al., 2022). Rogers et al. pointed out that 34.1% of patients with severe COVID-19 experienced memory impairment during the acute stage, but only 18.9% in the post-illness stage showed memory impairment (Rogers et al., 2020). Therefore, to promote fast recovery of memory function in COVID-19 patients, treatment options were listed in Table 4. Potential therapeutic targets investigated by past studies were also summarized in Table 4 for future research in this field.

**Table 4:** Recommended therapeutic regimens to tackle with memory impairment in COVID-19 patients.

1. Regular physical activity, both indoors and outdoors, has shown its benefits in improving memory function all the time, not excepting the pandemic era (Jimeno-Almazán et al., 2021).
2. Doing cognitive activities regularly has been shown to be effective in strengthening memory function (e.g., short-term memory) and limiting the negative effects of memory impairment due to COVID-19 (Wen et al., 2021; Cruz et al., 2022).
3. Adherence to a brain-boosting diet plays a critical role in contributing to memory performance during the pandemic (Sohel et al., 2021): (1) cut down on sugar in daily diet, as a long-term high sugar diet was shown to lead to poor memory and declined brain volume, especially in the brain regions associated with short-term memory (Hartmann et al., 2020); (2) refined carbohydrates also need to be monitored and restricted (Hawkins et al., 2018); (3) alcohol should be consumed in moderation, as binge drinking was shown to cause damage to the hippocampus (Drissi et al., 2020); (4) Omega-3 fatty acids supplementation was suggested to possess positive effects on short-term memory function (Kuelzow et al., 2016).
4. Socializing with other people regularly, whether virtually or physically, helps combat depression and stress during the pandemic, and thus has a protective role in preventing and improving memory symptoms (Cooper et al., 2021).
5. In addition to the evidence-based treatments above, the leaky RyR2 channels, HMGB1 and kynurenine pathway (KP) have been found to be potential therapeutic targets for improving COVID-19-related memory symptoms (Street, 2020; Cysique et al., 2022; Reiken et al., 2022).

**Figure 2:**
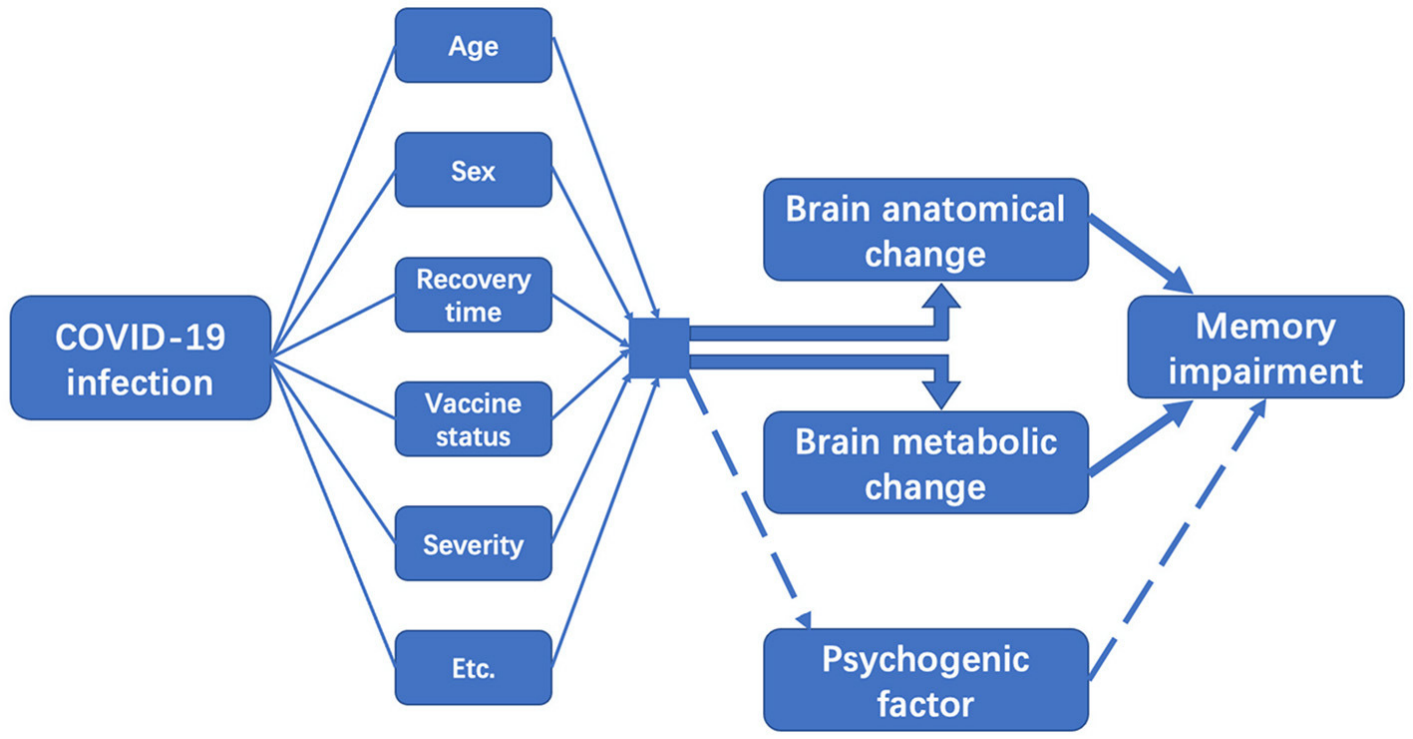
Schematic representation of memory impairment after COVID-19 infection.

Neuroimaging scans help to elucidate the COVID-19-related neuropathological changes in human brains, therefore providing avenues to explore memory impairment. According to the past evidence-based findings, although clinicoimaging correlations cannot prove causality between COVID-19 infection and memory deficits, we still argue that hypometabolism in certain brain regions, increased white matter hyperintensities and decline in cerebral gray matter volume may be effective predictors of the memory symptoms in COVID-19 patients, especially in the early stage of COVID-19 infection and those with persistent memory complaints. To assess such association, many other factors are still needed to be taken into consideration. Figure 2 is a schematic representation of memory impairment in patients following COVID-19 infection.

The available imaging evidence in this review has provided substantial support for interpreting the association between COVID-19-induced pathological changes in brain anatomy and metabolism and impaired memory performance in patients, but the type of memory impairment in patients remains obscure and therefore warrants further investigation. Future research focusing on a specific type of memory (e.g., working memory, procedural memory and episodic memory) is strongly encouraged, so as to narrow down the most obvious type of memory deficits caused by COVID-19, and thus find specialized treatment strategies to counteract them. Also, most of the included studies were conducted in the context of early variants of COVID-19, so very little research has specifically examined the long-term effects of the latest and most prevalent one—Omicron and its subvariants on memory performance. Hence, the recruitment of patients with recent COVID-19 infection for additional studies would be valuable. In addition, the vaccination status of subjects in early studies varied widely, in contrast to the current global COVID-19 vaccination coverage, which has been much higher than previously (Huang C. et al., 2022; Huang S. et al., 2022; McLaughlin et al., 2022). Since Ku et al. noted the protective effects of COVID-19 vaccine on brain after COVID-19 infection (Ku et al., 2021), patients' vaccination status might be an important factor to be considered while exploring this association in future studies.

To the best of our knowledge, this is the first systematic review study to present impacts of COVID-19 on human memory functions *via* neuroimaging evidence. We compared and contrasted past studies using neuroimaging of COVID-19-related memory dysfunction (Tables 2, 3). Some prior studies investigating patients' memory deficits in the absence of brain imaging methods were not included in this review, but they also provided ample evidence of memory impairment associated with COVID-19 infection (Bertuccelli et al., 2022). For instance, Reiken et al. provided evidence linking COVID-19 infection, cognitive impairment, and Alzheimer's-like signals shown in brain lysates of COVID-19 patients (Reiken et al., 2022).

## Limitation

The major limitation was about methodologic limitations of the original studies included. Specifically, there is a lack of a correlational analysis to appraise the relationship between brain pathological changes and memory impairment in patients. Also, over 40% of the studies included were identified as case studies or case series, which limited the validity and reliability of the perspectives in this review study. Moreover, we were only able to provide qualitative syntheses of the data due to the limited number of quantitative controlled neuroimaging studies. Thus, comprehensive meta-analyses studies and longitudinal investigation with correlational analyses are needed in further investigation.

## Conclusion

In conclusion, sufficient evidence shows that memory impairment is a prominent symptom of COVID-19, and is likely associated with COVID-19-induced brain dysfunction. We are beginning to understand the pathophysiology of COVID-19-related memory impairment. Hypometabolism, increased white matter hyperintensities, and decreased cerebral gray matter volume may be effective indicators of memory dysfunction in COVID-19 patients, but a causal relationship remains unproven. Further longitudinal investigations combined with correlational analyses are needed to better understand such correlation.

## Author contributions

DS, SL, and RX contributed to conception and design of the study. DS and SL conducted the protocol and the searches. RX, GN, and JH performed the screening. GN, JH, and ZD performed the data extraction and rating. DS wrote the first draft of the manuscript. YZ, ZX, and XG revised the first version of the manuscript. YX made a significant contribution to the latest manuscript. All authors contributed to the article and approved the submitted version.
